# Surgical resection significantly promotes the overall survival of patients with hepatocellular carcinoma: a propensity score matching analysis

**DOI:** 10.1186/s12876-021-01807-4

**Published:** 2021-05-14

**Authors:** Yaw-Sen Chen, Pei-Min Hsieh, Hung-Yu Lin, Chao-Ming Hung, Gin-Ho Lo, Yao-Chun Hsu, I.-Cheng Lu, Chih-Yuan Lee, Tsung-Chin Wu, Jen-Hao Yeh, Pojen Hsiao, Yu-Chan Li, Ya-Chin Wang, Kun-Chou Hsieh, Chih-Wen Lin

**Affiliations:** 1 Division of Gastroenterology and Hepatology, E-Da Dachang Hospital, I-Shou University, No. 1, Yida Road, Jiaosu Village, Yanchao District, Kaohsiung, 807 Taiwan, ROC; 2 Division of Gastroenterology and Hepatology, Department of Medicine, E-Da Hospital, I-Shou University, Kaohsiung, 824 Taiwan, ROC; 3 Health Examination Center, E-Da Hospital, I-Shou University, Kaohsiung, 824 Taiwan, ROC; 4 School of Medicine, College of Medicine, I-Shou University, Kaohsiung, 824 Taiwan, ROC; 5 Department of Surgery, E-Da Hospital, I-Shou University, Kaohsiung, 824 Taiwan, ROC; 6 School of Chinese Medicine, College of Chinese Medicine, China Medical University, Taichung, 404 Taiwan, ROC; 7 Research Center for Traditional Chinese Medicine, China Medical University Hospital, Taichung, 404 Taiwan, ROC; 8 Department of Surgery, National Taiwan University Hospital, Taipei, 100 Taiwan, ROC

**Keywords:** Surgical resection, Hepatocellular carcinoma, Overall survival, Hepatitis B virus, Prognosis

## Abstract

**Background:**

The benefits of surgical resection (SR) for various Barcelona Clinic Liver Cancer (BCLC) stages of hepatocellular carcinoma (HCC) remain unclear. We investigated the risk factors of overall survival (OS) and survival benefits of SR over nonsurgical treatments in patients with HCC of various BCLC stages.

**Methods:**

Overall, 2316 HCC patients were included, and their clinicopathological data and OS were recorded. OS was analyzed by the Kaplan-Meier method and Cox regression analysis. Propensity score matching (PSM) analysis was performed.

**Results:**

In total, 66 (2.8%), 865 (37.4%), 575 (24.8%) and 870 (35.0%) patients had BCLC stage 0, A, B, and C disease, respectively. Furthermore, 1302 (56.2%) of all patients, and 37 (56.9%), 472 (54.6%), 313 (54.4%) and 480 (59.3%) of patients with BCLC stage 0, A, B, and C disease, respectively, died. The median follow-up duration time was 20 (range 0–96) months for the total cohort and was subdivided into 52 (8–96), 32 (1–96), 19 (0–84), and 12 (0–79) months for BCLC stages 0, A, B, and C cohorts, respectively. The risk factors for OS were (1) SR and cirrhosis; (2) SR, cirrhosis, and Child–Pugh (C–P) class; (3) SR, hepatitis B virus (HBV) infection, and C–P class; and (4) SR, HBV infection, and C–P class for the BCLC stage 0, A, B, and C cohorts, respectively. Compared to non-SR treatment, SR resulted in significantly higher survival rates in all cohorts. The 5-year OS rates for SR vs. non-SR were 44.0% versus 28.7%, 72.2% versus 42.6%, 42.6% versus 36.2, 44.6% versus 23.5%, and 41.4% versus 15.3% (all *P* values < 0.05) in the total and BCLC stage 0, A, B, and C cohorts, respectively. After PSM, SR resulted in significantly higher survival rates compared to non-SR treatment in various BCLC stages.

**Conclusions:**

SR conferred significant survival benefits to patients with HCC of various BCLC stages and should be considered a recommended treatment for select HCC patients, especially patients with BCLC stage B and C disease.

**Supplementary Information:**

The online version contains supplementary material available at 10.1186/s12876-021-01807-4.

## Background

Hepatocellular carcinoma (HCC) is a major cause of cancer-related death worldwide [1]. HCC staging systems have been developed for treatment and prognostic evaluation [2–7]. The Barcelona Clinic Liver Cancer (BCLC) system is widely utilized because it incorporates tumor burden, liver cirrhosis severity, and patient performance status and is thus advantageous for treatment and prognostic assessment [4, 6, 7]. The BCLC system is approved by the American Association for the Study of Liver Disease (AASLD) and the European Association for the Study of Liver (EASL) [6, 7]. Patients with stage 0 (very early-stage) and stage A (early-stage) HCC are recommended to undergo surgical resection (SR), while patients with stage B (intermediate-stage) and stage C (advanced-stage) HCC are recommended to undergo transcatheter arterial chemoembolization (TACE) and sorafenib treatment according to the BCLC system. However, the BCLC system is limited because of differences in tumor conditions and heterogeneity in the prognosis of various stages of disease, especially BCLC stages B and C [8, 9]. Recently, numerous studies, mostly from Asia-Pacific countries, have focused on increasing the use of SR in patients with BCLC stage B and C disease and have demonstrated better overall survival (OS) in patients who have undergone SR as compared to patients with nonsurgical treatments [9–13]. However, some studies have shown that TACE is not inferior to SR for patients with operable BCLC stage B and C HCC [14]. The advantages of SR over nonsurgical treatments for HCC of various BCLC stages are still unknown. Furthermore, several prognostic factors, including age, treatment, liver function, tumor size, and etiology, are associated with OS in HCC patients [15, 16]. However, the prognostic factors for survival in HCC patients remain elusive. This study aimed to investigate the risk factors of OS and the potential benefits of SR over nonsurgical treatments in a large cohort of HCC patients.

## Methods

### Patients and follow-up

**Fig. 1:**
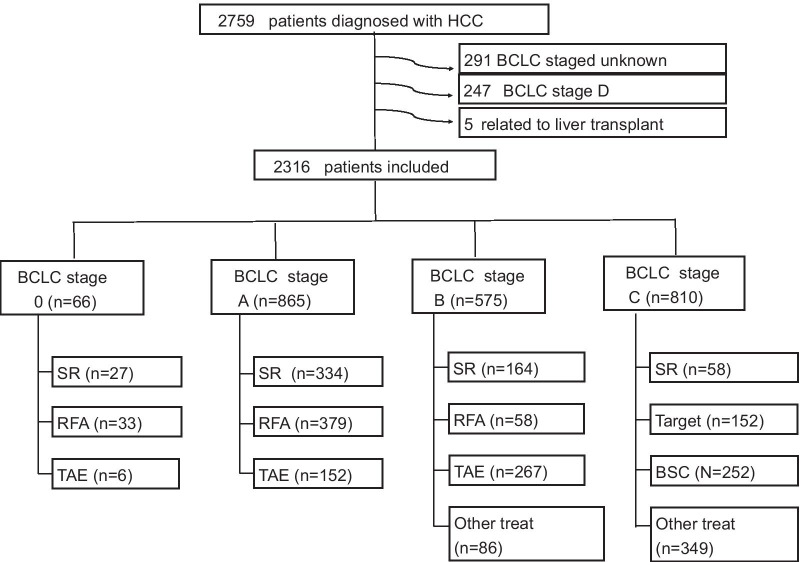
Study flowchart and inclusion of participants

We retrospectively collected information on 2759 patients diagnosed with HCC between 2010 and 2016 at E-Da Hospital, I-Shou University, Kaohsiung, Taiwan and 543 patients were excluded (Fig. 1). The study was conducted in accordance with the guidelines of the International Conference on Harmonization for Good Clinical Practice and was approved by the Ethics Committee of E-Da Hospital, I-Shou University (EMRP-107-130). Patients were diagnosed with HCC based on histological confirmation or at least one typical imaging method according to the recommendations of the AASLD [6]. OS was defined as the time from the date of diagnosis to the date of death or last follow-up, and the last follow-up was in December 2017. SR was defined as hepatic resection for HCC. Non-SR treatments included radiofrequency ablation (RFA), TACE, hepatic artery infusion chemotherapy (HAIC), targeted therapy (sorafenib), radiotherapy (RT) and best supportive care (BSC). Patients with older age received RFA or TACE in BCLC stage 0 and A because patients refused SR due to the possibility of older age or high risk for post-operative morbidity and mortality. Patients underwent SR in BCLC stage B because of patients with resectable HCC lesions and indocyanine green is less than 10%. Patients underwent SR in BCLC stage C because patients were resectable HCC lesions and indocyanine green is less than 10%. Clinicographic data, smoking, excessive alcohol use, hepatitis status, liver cirrhosis, Child–Pugh (C–P) class, tumor size, tumor number, and vascular invasion, were examined. Tumor number, tumor size and vascular invasion were mostly determined based on radiologic findings and confirmed by pathologic findings if appropriate. Liver cirrhosis was diagnosed based on pathologic findings and/or evaluated by ultrasound, computed tomography, or magnetic resonance imaging. The functional status of the liver was evaluated using the C–P scoring system[17].

### Data analysis and statistics

All statistical analyses were performed using SPSS ver. 18.0 (SPSS, Chicago, IL, USA). Numerical data were expressed as medians and ranges. Categorical data were described using numbers and percentages. OS was determined using the Kaplan-Meier method and compared among patients with HCC of various BCLC stages and among patients receiving different treatments. Cox proportional hazards regression analysis of OS in HCC patients was performed according to BCLC stages. Variables including sex, age, smoking, alcohol use, HBV infection, hepatitis C virus (HCV) infection, cirrhosis, C–P class, tumor size, tumor number, and treatment were incorporated into the Cox regression analysis. Moreover, we used logistic regression to generate propensity score matching (PSM) with with sex, age, cirrhosis, C–P class, tumor size, and tumor number for all patients of various BCLC stage in order to reduce bias in our analyses. The two treatment groups were matched with the control group according to the generated PSM using a caliper width of 0.02. On the completion of matching, the baseline covariates were compared using the paired t-test or Mann–Whitney U test for continuous variables and the chi-square test for categorical variables. A *P* value < 0.05 was used to determine statistical significance.

## Results

### Baseline demographic data

A total of 2316 HCC patients were included in this study (Fig. 1). The demographic and clinicopathological features of the 2316 patients (75.5% male, median age of 63 years) are shown in Table 1. Regarding the etiology of HCC, 71.6% of the patients had HBV infection, 30.6% had HCV infection, and 37.5% had excessive alcohol use. Approximately 37.9% of patients had liver cirrhosis, and of those patients, 60.8% had C–P class A disease. The mean tumor size was 6.1 cm and the mean tumor number was 2.1 tumors. Moreover, 1302 patients (56.2%) were mortality and the median follow-up time was 22 (range, 1–96) months.

### Overall survival of patients in the total and various BCLC stage cohorts

**Fig. 2:**
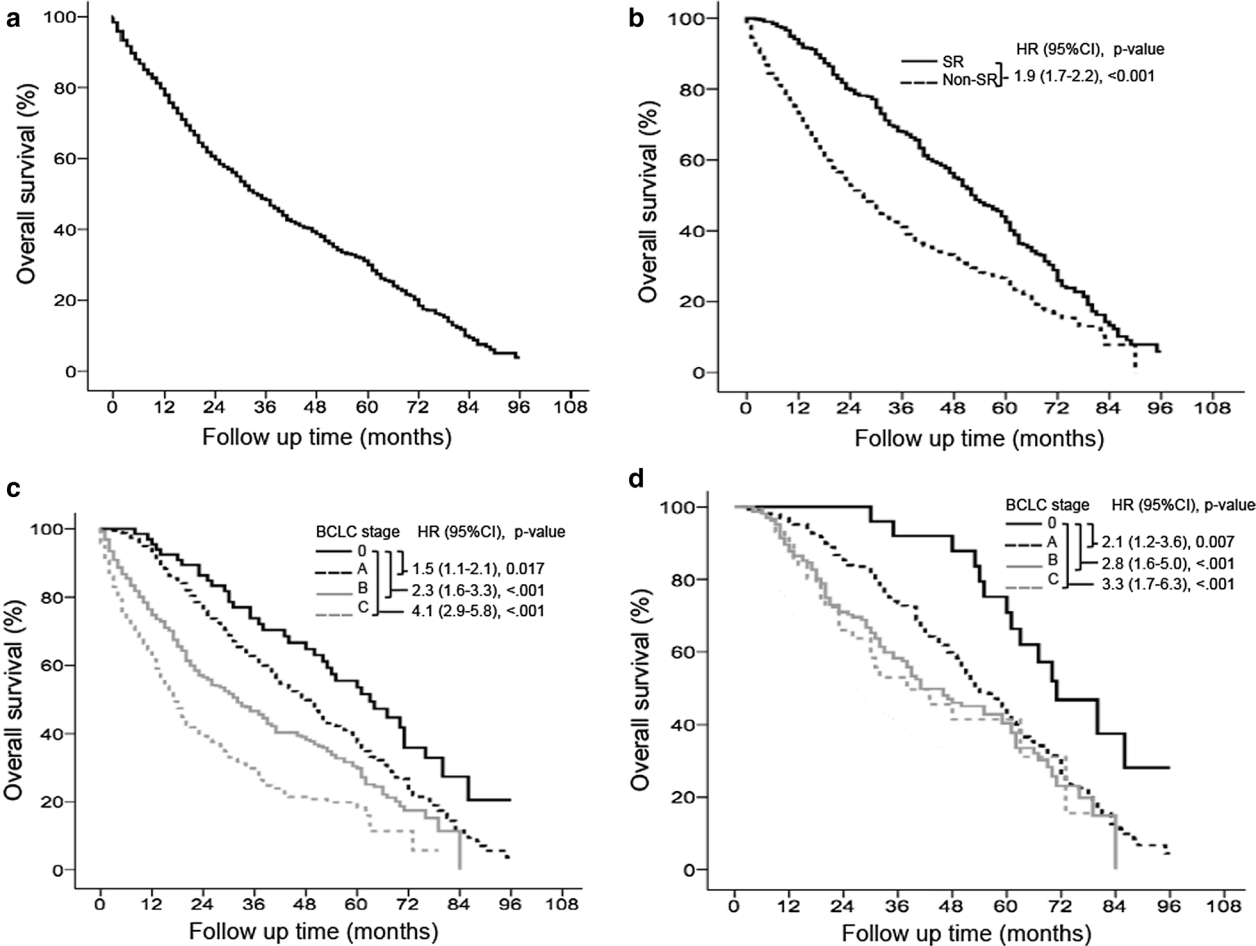
Overall survival in patients with BCLC stage B and C disease. Surgical resection (SR) resulted in significantly higher overall survival (OS) rates than non-SR treatments in BCLC stage B (*P* < 0.05) (**a**). After propensity score matching (PSM), SR still had significantly higher OS rates than non-SR treatments in BCLC stage B (*P* < 0.05) (**b**). SR resulted in significantly higher OS rates than non-SR treatments in BCLC stage C (*P* < 0.05) (**c**). After PSM, SR still had significantly higher OS rates than non-SR treatments in BCLC stage C (*P* < 0.05) (**d**)

Of the 2316 patients, 1302 (56.2%) died, and the median follow-up duration was 20 (range, 1–96) months (Table 1). The mortality rate was 35.5% per person-year. The cumulative OS rates at 5 years were 32.5% (Fig. 2a). SR was performed in 538 (23.2%) patients, and the OS was significantly better in these patients than in non-SR patients. The cumulative OS rates at 5 years in the SR and non-SR groups were 44.0 and 28.7%, respectively (*P* < 0.001, Fig. 2b). Survival was significantly higher in the BCLC stage 0 cohort than in the BCLC stage A, B, and C cohorts (*P* < 0.05). The cumulative OS rates at 5 years in the BCLC stage 0, A, B, and C cohorts were 59.5%, 38.7%, 31.6 and 23.4%, respectively (Fig. 2c). For patients receiving SR, survival was significantly higher in the BCLC stage 0 cohort than in the BCLC stage A, B, and C cohorts (*P* < 0.01). The cumulative OS rates for SR patients at 5 years in the BCLC stage 0, A, B, and C cohorts were 72.2%, 42.6%, 44.6 and 41.4%, respectively (Fig. 2d).

**Table 1:** Basic demographic data of patients with hepatocellular carcinoma of different BCLC stages

Variable	Total (N = 2316)		BCLC stage 0 (N = 66)	BCLC stage A (N = 865)	BCLC stage B (N = 575)	BCLC stage C (N = 810)
Male	1748 (75.5)		46 (69.7)	605 (69.6)	437 (76.0)	660 (81.5)
Age (years)	63 (19–99)		62 (23–85)	63 (28–92)	64 (30–91)	61 (19–99)
Smoking	949 (41.0)		28 (42.4)	316 (36.5)	232 (40.3)	373 (46.0)
Alcohol use	868 (37.5)		22 (33.3)	283 (32.7)	218 (37.9)	345 (42.6)
HBV positive	1658 (71.6)		44 (66.7)	599 (69.2)	414 (72.0)	601 (74.2)
HCV positive	696 (30.1)		14 (21.2)	253 (29.2)	172 (29.9)	257 (31.7)
Cirrhosis	1395 (60.2)		43 (65.2)	511 (59.1)	334 (58.1)	507 (62.6)
Child–Pugh class A	877 (37.9)		66 (100)	277 (32.0)	225 (39.1)	309 (38.1)
Tumor size (cm)	6.1 (1.0–27)		1.7 (1.0–2.0)	2.9 (1.0–5.0)	7.5 (1.2–21)	9.4 (1.2–27)
Tumor number	2.1 (1–11)		1 (1–1)	1.5 (1–3)	2.5 (1–11)	2.7 (1–11)
Treatment, surgery	538 (23.2)		27 (40.9)	289 (33.4)	164 (28.5)	58 (7.2)
Mortality	1302 (56.2)		37 (56.9)	472 (54.6)	313 (54.4)	480 (59.3)
Median follow-up duration (months)	20 (0–96)		52 (8–96)	32 (1–96)	19 (0–84)	12 (0–79)

BCLC stage: Barcelona clinic liver cancer; HBV: Hepatitis B virus; HCV: Hepatitis C virus; OS: Overall survival; Number (%); Mean (range)

### Overall survival of patients with BCLC stage 0 disease

**Fig. 3:**
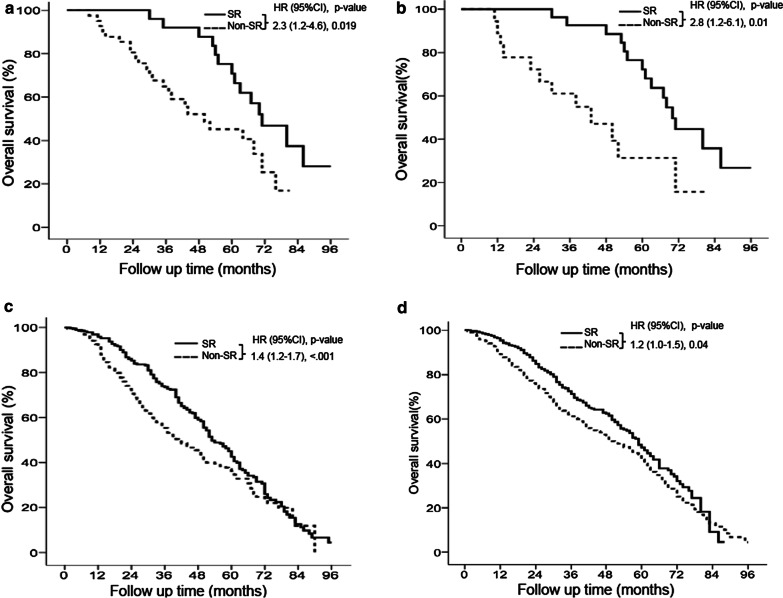
Overall survival of total cohort in various BCLC stages. Overall survival (OS) of total cohort (n = 2316) (**a**). Surgical resection (SR) resulted in significantly higher OS rates than non-SR treatments in all 2316 HCC patients (*P* < 0.05) (**b**). OS rates based on Cox regression analysis in patients with HCC of various BCLC stages. Patients with stage 0 disease had significantly better OS than patients stage A, B, and C disease (**c**). OS rates based on Cox regression analysis in patients with HCC of various BCLC stages undergoing SR. SR resulted in significantly better OS in patients with stage 0 than in patients with stage A, B, and C disease (**d**)

Among the 66 HCC patients with BCLC stage 0 disease, 37 (56.9%) died, and the median follow-up duration was 52 (range, 8–96) months (Table 1). For the multivariate analysis, Cox proportional hazards modeling showed that patients with cirrhosis were associated with lower survival compared to patients without cirrhosis (hazard ratio [HR]: 0.60; 95% confidence interval [CI]: 0.42–0.72, *P* = 0.006), while patients undergoing SR were associated with higher survival compared to patients without SR (HR: 2.31; 95% CI: 1.22–4.61, *P* = 0.019) (Table 2). There are significant differences in age and HBV infection between SR and non-SR treatments in Table 3. Twenty-seven (40.9%) patients underwent SR and 39 (59.1%) received non-SR treatments. Patients undergoing SR had significantly higher survival rates than patients receiving nonsurgical treatment (*P* = 0.019), RFA (HR: 2.1; 95% CI: 1.1–4.4, *P* = 0.037), or TACE (HR: 4.3; 95% CI: 1.3–13, *P* = 0.015) (Fig. 3a and Additional file 1: Fig. S1A). The cumulative OS rates at 5 years in the SR and non-SR treatments were 72.2 and 42.6%, respectively (Fig. 3a). The basic demographic data of patients with HCC of various BCLC stages between SR and non-SR treatments after PSM was not significant difference and was shown in Table 4. After PSM, patients undergoing SR had significantly higher survival rates than patients receiving nonsurgical treatment (*P* = 0.01). The cumulative OS rates at 5 years in the SR and non-SR treatments were 72.2 and 31.4%, respectively (Fig. 3b).

**Table 2:** Cox regression analysis of overall survival in patients with hepatocellular carcinoma of different BCLC stages

Variable	BCLC stage 0 (N = 66)		BCLC stage A (N = 865)		BCLC stage B (N = 575)		BCLC stage C (N = 810)	
	HR (95 % CI)	*P* value	HR (95 % CI)	*P* value	HR (95 % CI)	*P* value	HR (95 % CI)	*P* value
Sex								
Female	1		1		1		1	
Male	0.70 (0.85–1.98)	0.79	0.81 (0.64–1.03)	0.09	0.95 (0.68–1.31)	0.76	0.90 (0.67–1.21)	0.49
Age (years)								
< 60	1		1		1		1	
≥ 60	0.83 (0.61–1.11)	0.22	0.84 (0.64–1.09)	0.19	0.71 (0.50–1.01)	0.06	0.81 (0.59–1.09)	0.17
Smoking								
Yes	1		1		1		1	
No	0.52 (0.22–1.23)	0.14	0.59 (0.08–4.41)	0.61	0.89 (0.37–2.20)	0.82	0.81 (0.30–2.19)	0.68
Alcohol use								
Yes	1		1		1		1	
No	0.70 (0.10–6.25)	0.67	0.75 (0.10–5.59)	0.78	0.82 (0.55–1.22)	0.33	0.52 (0.19–1.42)	0.20
HBV								
Negative	1		1		1		1	
Positive	0.30 (0.04–1.21)	0.25	0.22 (0.03–1.62)	0.14	0.43 (0.27–0.71)	0.001	0.40 (0.27–0.58)	< 0.001
HCV								
Negative	1		1				1	
Positive	0.95 (0.73–1.10)	0.49	0.51 (0.07–3.78)	0.51	0.88 (0.66–1.18)	0.41	0.89 (0.41–1.12)	0.35
Cirrhosis								
Absent	1		1		1		1	
Present	0.60 (0.42–0.72)	0.006	0.55 (0.39–0.78)	0.001	0.91 (0.59–1.40)	0.66	0.87 (0.53–1.43)	0.58
Child-Pugh class								
A			1		1		1	
B			0.55 (0.43–0.71)	< 0.001	0.41 (0.28–0.61)	< 0.001	0.40 (0.29–0.55)	< 0.001
Tumor size	0.98 (0.97–1.01)	0.238	1.06 (0.94–1.20)	0.289	1.03 (0.92–1.15)	0.609	0.98 (0.96–1.01)	0.238
Tumor number	0.95 (0.96–1.02)	0.058	1.01 (0.83–1.20)	0.971	1.06 (0.91–1.24)	0.391	0.94 (0.94–1.05)	0.052
Treatment								
Non-surgery	1		1		1		1	
Surgery	2.31 (1.22–4.61)	0.019	1.41 (1.17–1.70)	< 0.001	2.10 (1.56–2.82)	< 0.001	3.10 (2.02–4.70)	< 0.001

BCLC stage: Barcelona clinic liver cancer; HR: Hazard ratio; CI: Conference incidence; HBV: Hepatitis B virus; HCV: Hepatitis C virus

**Table 3:** Basic demographic data of patients with hepatocellular carcinoma of different BCLC stages between surgical resection and non-surgical resection

Variable	BCLC stage 0 (N = 66)			BCLC stage A (N = 865)			BCLC stage B (N = 575)			BCLC stage C (N = 810)		
	SR (N = 27)	Non-SR (n = 39)	*P *value	SR (N = 289)	Non-SR (N = 576)	*P *value	SR (N = 164)	Non-SR (N = 411)	*P *value	SR (N = 58)	Non-SR (N = 752)	*P* value
Male	21 (77.8)	25 (64.1)	0.235	225 (77.9)	380 (66)	0.001	135 (82.3)	302 (73.5)	0.025	45 (77.6)	615 (81.8)	0.428
Age (years)	55 (23–83)	66 (24–85)	0.001	59 (29–90)	63 (28–92)	0.0001	63 (30–88)	64 (30–91)	0.095	60 (25–87)	61 (19–99)	0.597
Smoking	10 (37)	18 (46.2)	0.461	92 (31.8)	224 (38.9)	0.042	71 (43.3)	161 (39.2)	0.363	20 (34.5)	353 (46.9)	0.067
Alcohol use	8 (29.6)	14 (35.9)	0.595	84 (29.1)	199 (34.5)	0.105	67 (40.9)	151 (36.7)	0.359	17 (29.3)	328 (43.6)	0.034
HBV positive	23 (85.2)	21 (53.8)	0.008	197 (68.2)	402 (69.8)	0.625	111 (67.7)	303 (73.7)	0.145	41 (70.7)	560 (74.5)	0.526
HCV positive	5 (18.5)	9 (23.1)	0.656	81 (28)	172 (29.9)	0.576	41 (25)	131 (31.9)	0.104	18 (31)	239 (31.8)	0.906
Cirrhosis	20 (74.1)	23 (59)	0.206	172 (59.5)	339 (58.9)	0.852	95 (57.9)	239 (58.2)	0.961	28 (48.3)	479 (63.7)	0.019
Child-Pugh class A	20 (74.1)	23 (59)	0.206	87 (30.1)	190 (33.0)	0.212	60 (36.6)	165 (40.1)	0.327	20 (34.5)	289 (38.4)	0.109
Tumor size (cm)	1.7 (1.0–2.0)	1.6 (1.0–2.0)	0.546	3.1 (1.0–5.0)	2.7 (1.0–5.0)	< 0.001	8.1 (1.2–21)	7.3 (1.2–21)	0.002	9.5 (1.2–27)	9.4 (1.2–27)	0.850
Tumor number	1 (1–1)	1 (1–1)	1.000	1.4 (1–3)	1.5 (1–3)	0.089	2.1 (1–5)	2.6 (1–11)	0.003	2.7 (1–11)	2.7 (1–11)	0.890
Mortality	14 (51.9)	23 (58.9)	0.566	193 (66.80	279 (48.4)	0.0001	158 (60.1)	293 (48.7)	0.002	28 (48.3)	452 (60.1)	0.077
Median OS (months)	71 (30–96)	44 (8–81)		53 (3–88)	41 (1–96)		41 (4–84)	24 (1–76)		38 (6–79)	16 (1–66)	

BCLC stage: Barcelona clinic liver cancer; SR: Surgical resection; HR: Hazard ratio; CI: Conference incidence; HBV: Hepatitis B virus; HCV: Hepatitis C virus; OS: Overall survival; Number (%); Mean or Median (range)

**Table 4:** Basic demographic data of patients with hepatocellular carcinoma of different BCLC stages between surgical resection and non-surgical resection after propensity score matching

Variable	BCLC stage 0 (N = 43)			BCLC stage A (N = 578)			BCLC stage B (N = 327)			BCLC stage C (N = 116)		
	SR (N = 27)	Non-SR (N = 18)	*P* value	SR (N = 289)	Non-SR (N = 289)	*P* value	SR (N = 164)	Non-SR (N = 163)	*P* value	SR (N = 58)	Non-SR (N = 58)	*P* value
Male	21 (77.8)	13 (72.2)	0.671	208 (72)	197 (68.2)	0.318	135 (82.3)	137 (84)	0.675	45 (77.6)	48 (82.8)	0.485
Age (years)	55 (23–83)	59 (24–85)	0.312	62 (29–90)	60 (28–92)	0.066	61 (30–88)	62 (30–91)	0.346	60 (25–87)	60 (19–99)	0.910
Smoking	10 (37)	10 (55.6)	0.221	114 (39.4)	95 (32.9)	0.128	71 (43.3)	57 (35.0)	0.123	20 (34.5)	16 (27.5)	0.151
Alcohol use	8 (29.8)	7 (38.9)	0.519	96 (33.2)	86 (29.8)	0.371	67 (40.9)	62 (38.0)	0.602	17 (29.3)	13 (22.4)	0.283
HBV positive	25 (92.6)	12 (66.7)	0.260	200 (69.2)	200 (69.2)	1.000	111 (67.7)	108 (66.3)	0.784	41 (70.7)	36 (62.1)	0.085
HCV positive	6 (22.2)	5 (27.5)	0.671	89 (30.8)	76 (26.3)	0.231	41 (25.0)	44 (27.0)	0.681	18 (31.0)	11 (19.0)	0.133
Cirrhosis	20 (74.1)	13 (72.2)	0.891	177 (61.2)	168 (58.1)	0.445	95 (57.9)	80 (49.1)	0.109	28 (48.3)	27 (46.5)	0.873
Child–Pugh class A	20 (74.1)	13 (72.2)	0.891	87 (30.1)	92 (31.8)	0.819	60 (36.6)	68 (41.7)	0.198	20 (34.5)	18 (31.0)	0.796
Tumor size (cm)	1.7 (1.0–2.0)	1.6 (1.0–2.0)	0.812	2.8 (1.0–5.0)	2.7 (1.0–5.0)	0.289	7.7 (1.2–21)	7.5 (1.2–21)	0.105	9.5 (1.2–27)	9.4 (1.2–27)	0.885
Tumor number	1 (1–1)	1 (1–1)	1.000	1.4 (1–3)	1.5 (1–3)	0.227	2.3 (1–5)	2.5 (1–11)	0.077	2.7 (1–11)	2.7 (1–11)	0.903
Mortality	15 (55.6)	12 (66.7)	0.456	155 (53.6)	183 (63.3)	0.018	95 (57.9)	103 (63.2)	0.33	28 (48.3)	55 (94.8)	0.0001
Median OS (months)	70 (30–96)	43 (11–81)		53 (3–88)	46 (1–96)		41 (4–84)	20 (1–72)		38(6–79)	16 (1–62)	

BCLC stage: Barcelona clinic liver cancer; SR: Surgical resection; HR: Hazard ratio; CI: Conference incidence; HBV: Hepatitis B virus; HCV: Hepatitis C virus; OS: Overall survival; Number (%); Mean or Median (range)

### Overall survival of patients with BCLC stage A disease

Among the 865 HCC patients with BCLC stage A disease, 472 (54.6%) died, and the median follow-up duration was 32 (range, 1–96) months (Table 1). For the multivariate analysis, patients with cirrhosis and C–P class B were associated with lower survival compared to patients without cirrhosis (HR: 0.55; 95% CI: 0.39–0.78, *P* = 0.001) and patients with C–P class A (HR: 0.55; 95% CI: 0.43–0.71, *P* < 0.001), respectively. Additionally, patients undergoing SR were associated with higher survival compared to patients without SR (HR: 1.41; 95% CI: 1.17–1.70, *P* < 0.0001) (Table 2). There are significant differences in sex, age, smoking, and tumor number between SR and non-SR treatments in Table 3. Besides, 334 (38.6%) patients underwent SR, 531 (61.4%) received non-SR treatments. Patients undergoing SR had significantly higher survival rates than patients receiving non-SR treatments (*P* < 0.0001), RFA (HR: 1.2; 95% CI: 1.1–1.5, *P* = 0.041), or TACE (HR: 1.7; 95% CI: 1.3–2.2, *P* < 0.001) (Fig. 3c and Additional file 1: Fig. S1B). The cumulative OS rates at 5 years in the SR and non-SR treatments were 42.6 and 36.2%, respectively (Fig. 3c). After PSM, patients undergoing SR had significantly higher survival rates than patients receiving non-SR treatments (*P* = 0.04). The cumulative OS rates at 5 years in the SR and non-SR treatments were 42.6 and 41.8%, respectively (Fig. 3d).

### Overall survival of patients with BCLC stage B disease

**Fig. 4:**
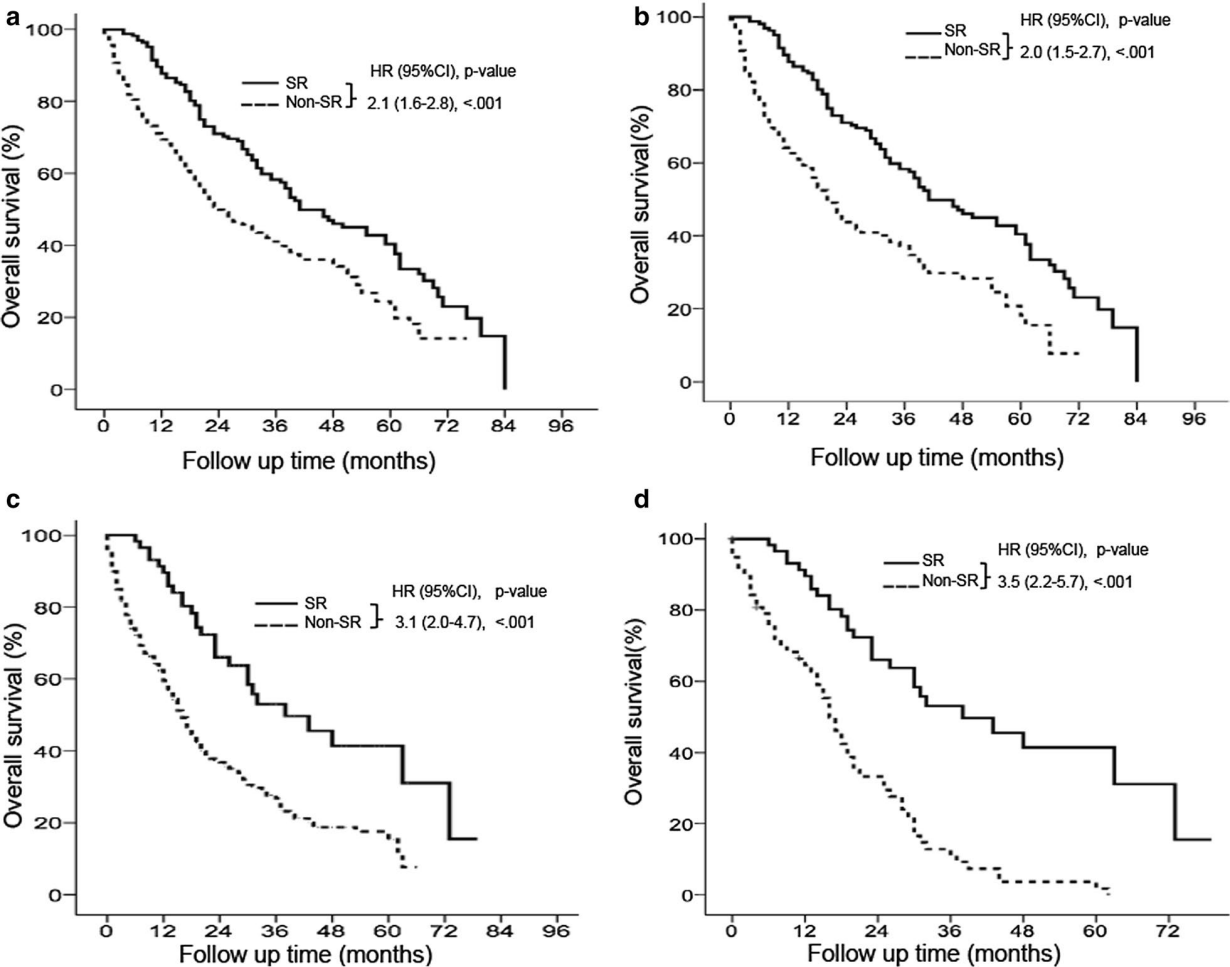
Overall survival in patients with BCLC stage 0 and A disease. Surgical resection (SR) resulted in significantly higher overall survival (OS) rates than non-SR treatments in BCLC stage 0 (*P* < 0.05) (**a**). After propensity score matching (PSM), SR still had significantly higher OS rates than non-SR treatments in BCLC stage 0 (*P* < 0.05) (**b**). SR resulted in significantly higher OS rates than non-SR treatments in BCLC stage A (*P* < 0.05) (**c**). After PSM, SR still had significantly higher OS rates than non-SR treatments in BCLC stage A (*P* < 0.05) (**d**)

Among the 575 HCC patients with BCLC stage B disease, 313 (54.4%) died, and the median follow-up duration was 19 (range, 1–84) months (Table 1). For the multivariate analysis, patients with HBV infection and C–P class B were associated with lower survival compared to patients without HBV infection (HR: 0.43; 95% CI: 0.27–0.71, *P* = 0.001) and patients with C–P class A (HR: 0.41; 95% CI: 0.28–0.61, *P* < 0.001), respectively. Patients undergoing SR were associated with higher survival compared to patients without SR (HR: 2.10; 95% CI: 1.56–2.82, *P* < 0.001) (Table 2). There are significant differences in sex and tumor size between SR and non-SR treatments in Table 3. In addition, 164 (28.5%) patients underwent SR and 411 (71.5%) received non-SR treatments. Patients undergoing SR had significantly higher survival rates than patients receiving non-SR treatments (*P* < 0.0001), RFA (HR: 1.4; 95% CI: 1.1–2.2, *P* = 0.043), TACE (HR: 1.7; 95% CI: 1.3–2.2, *P* < 0.001), or other treatments (HR: 2.3; 95% CI: 1.7–3.3, *P* < 0.001) (Fig. 4a and Additional file 1: Fig. S1C). The cumulative OS rates at 5 years in the SR and non-SR treatments were 40.4 and 23.5 %, respectively (Fig. 4a). After PSM, patients undergoing SR had significantly higher survival rates than patients receiving non-SR treatments (*P* < 0.001). The cumulative OS rates at 5 years in the SR and non-SR treatments were 40.4 and 18.2 %, respectively (Fig. 4b).

### Overall survival of patients with BCLC stage C disease

Among the 810 HCC patients with BCLC stage C disease, 313 (54.4%) died, and the median follow-up duration was 12 (range, 1–79) months (Table 1). For the multivariate analysis, patients with HBV infection and C–P class B were associated with lower survival compared to patients without HBV infection (HR: 0.40; 95% CI: 0.27–0.58, *P* < 0.001) and patients with C–P class A (HR: 0.40; 95% CI: 0.0.29–0.55, *P* < 0.001), respectively. Patients undergoing SR were associated with higher survival compared to patients without SR (HR: 3.10; 95% CI: 2.02–4.70, *P* < 0.001) (Table 2). There are significant differences in alcohol use and cirrhosis between SR and non-SR treatments in Table 3. Fifty-eight (7.2%) patients underwent SR and 752 (92.8%) patients received non-SR treatments. Patients undergoing SR had significantly higher survival rates than patients receiving non-SR treatments, RFA, TACE, target therapy, RT, HAIC, and other treatments (all *P* < 0.05) (Fig. 4c and Additional file 1: Fig. S1D). The cumulative OS rates at 5 years in the SR and non-SR treatments were 41.4 and 15.3%, respectively (Fig. 4c). After PSM, patients undergoing SR had significantly higher survival rates than patients receiving non-SR treatments (*P* < 0.001). The cumulative OS rates at 5 years in the SR and non-SR treatment groups were 41.4 and 1.8%, respectively (Fig. 4d).

## Discussion

In this large cohort study, we analyzed 2316 HCC patients to identify the prognostic factors and treatments affecting OS. Our results demonstrated that the risk factors for OS were SR, cirrhosis, C–P class, and HBV infection within groups with different BCLC stages (Table 2). SR resulted in significantly higher OS rates than non-SR treatments among patients with HCC of various BCLC stages. After PSM, SR still had significantly higher OS rates than non-SR treatments among patients with HCC of various BCLC stages. As SR conferred significant survival benefits to patients with HCC of different BCLC stages, it should be considered a recommended treatment for selected HCC patients, especially patients with BCLC stage B and C disease.

SR, RFA and liver transplantation are the recommended treatment modalities for very early- and early-stage HCC [6, 7]. Several studies have shown that SR results in better long-term OS than RFA in very early- and early-stage HCC [18–20]. Consistent with previous studies [18–20], our results demonstrated that SR resulted in significantly higher OS rates when compared to non-SR treatments especial in RFA treatment in patients with BCLC stage 0 and A disease.

TACE is recommended as a standard of care for the treatment of patients with BCLC stage B disease [6, 7]. Several HCC experts have proposed four substages based on the Eastern Cooperative Oncology Group (ECOG) performance, C–P class, and “up-to-7” criteria within BCLC stage B disease [21]. However, these criteria mostly indicate benefits from TACE. Based on the great improvements in surgical techniques and perioperative care, some treatments may not be suitable for patients with intermediate- and advanced-BCLC stage HCC. Our results showed that SR resulted in a significantly higher OS rate than non-SR Treatments including RFA, TACE, and other treatments in patients with BCLC stage B disease. Similarly, several studies from both Western and Eastern countries have demonstrated that SR results in higher long-term survival than non-SR treatments, even for patients with multiple tumors [9, 10, 13, 22]. Furthermore, compared with TACE, SR significantly increases survival in select patients with BCLC stage B HCC [13, 23]. SR is a safe and effective therapy for select patients with resectable multiple or large HCC lesions in the same half-liver and sufficient liver reserve. Hence, SR may be considered for select patients who fit these criteria and could be recommended for patients with BCLC stage B disease.

Patients with BCLC stage C disease have poor outcomes because of the presence of advanced HCC associated with major vascular invasion and/or extrahepatic metastasis. Sorafenib is the only recommended standard of care for advanced HCC based on the BCLC staging system. However, because of the large heterogeneity in the population with advanced-stage HCC, SR is no longer contraindicated and provides survival benefit [10, 22]. Moreover, several studies have demonstrated significantly favorable survival in HCC patients with major vascular invasion, including the portal vein, hepatic vein and inferior vena cava, after SR [24–26]. Our results also confirmed the data from previous studies [10, 22, 24] and demonstrated that SR improved OS rates in patients with advanced-stage HCC. Therefore, meticulous and accurate selection criteria (HCC is located on the left or right lobe of liver, and portal vein tumor thrombosis in the segmental branch or first branch of portal vein can be excised in the same half-liver) should be established to identify individuals, among patients with vascular invasion, who would benefit most from SR. Hence, SR may also be considered for select patients with BCLC stage C HCC.

Liver function preservation, including C–P class and cirrhosis, is an important non-oncological factor affecting OS. Poor liver function preservation decreases the efficacy of treatment and increases mortality. Our results showed that cirrhosis and C–P class significantly affect OS in patients with HCC of various BCLC stages. Patients with cirrhosis easily develop portal hypertension, liver failure, and HCC. Additionally, patients with C–P class B disease have low survival. It is important to treat liver disease using antiviral therapy and prevent liver disease progression.

Taiwan is a hyperendemic area for HBV-related liver diseases and HCC. HBV infection can result in hepatocarcinogenesis, and multiple mechanisms have been proposed, including the accumulation of genetic damage due to the induction of oxidative stress and immune-mediated hepatic inflammation. The integration of HBV DNA into the human genome occurs during the early steps of carcinogenesis and can induce alterations in cancer-related gene expression and chromosomal instability [27, 28]. Our study demonstrated that 71.6% of HCC patients had HBV infection and that HBV infection significantly reduced OS rates in patients with BCLC stage B and C disease. In Taiwan, HBV-related HCC accounted for 88% of all cases before 1990, whereas from 1990 to 2000, the proportion of HBV-related HCC decreased to 66% [29, 30]. Our study demonstrated that 71.6% of HCC patients had HBV infection, and the proportion of HBV-related HCC remained high in southern Taiwan. In addition, HBV infection significantly reduces OS rates in patients with BCLC stage B and C disease. Therefore, it is probable that HCC is caused not only by cirrhosis but also by HBV infection-induced hepatocarcinogenesis.

Our study has several limitations. First, as with all retrospective studies, there was some selection bias, including differences among patients regarding treatment decisions and the presence of incomplete data including alpha-fetoprotein, vascular invasion, extrahepatic metastases, performance status, and clinically relevant portal hypertension. Second, patients might receive multimodal treatments in a sequential manner, which would make direct comparison of every single treatment difficult in intermediate- and advanced-stage disease. Third, patients undergoing liver transplantation were not included because of the small sample size. Fourth, the concept of therapeutic hierarchy using the inverse probability of treatment weights and ITA.LI.CA staging will be further studied [31, 32].

## Conclusions

Compared with nonsurgical treatments, SR significantly promoted survival benefits not only in very early- and early-stage but also in intermediate- and advanced-BCLC stage HCC. These results are valid in the cohorts with propensity score matching, and does not always represent results for all patients with intermediate- and advanced-BCLC stage HCC. More effort should be made to determine the proper selection criteria for SR in patients, especially in patients with intermediate- and advanced-stage disease. Additionally, the BCLC staging system should be further modified based on results from the clinic and responses to combinations of various treatment modalities.

## Supplementary Information

**Additional file 1: Figure S1**. Overall survival in patients with BCLC stage 0, A, B, and C disease by Kaplan-Meier analysis. Surgical resection (SR) resulted in significantly higher overall survival than radiofrequency ablation (RFA) and transcatheter arterial chemoembolization (TACE) in BCLC stage 0 (*P* <0.05) (A). SR resulted in significantly higher overall survival than RFA and TACE in BCLC stage A (*P* <0.05) (B). SR resulted in significantly higher overall survival than RFA and TACE in BCLC stage A (*P* <0.05) (B). SR resulted in significantly higher overall survival than RFA, TACE, and other treatment in BCLC stage B (*PP* <0.05) (C). SR resulted in significantly higher overall survival than RFA, TACE, target therapy, radiotherapy (RTO), hepatic artery infusion therapy (HAIC), and best support care (BSC) in BCLC stage C (*P* <0.05) (D).

## Data Availability

Data is available from the corresponding author upon reasonable request.

## Abbreviations

HCC: Hepatocellular carcinoma
BCLC: Barcelona Clinic Liver Cancer
AASLD: American Association for the Study of Liver Disease
ESAS: European Association for the Study of Liver
SR: Surgical resection
TACE: Transcatheter arterial chemoembolization
OS: Overall survival
RFA: Radiofrequency ablation
HAIC: Hepatic artery infusion chemotherapy
RT: Radiotherapy
BSC: Best supportive care
C–P class: Child–Pugh class
HBV: Hepatitis B virus
HCV: Hepatitis C virus
PSM: Propensity score matching
CI: Confidence interval
HR: Hazard ratio
ECOG: Eastern Cooperative Oncology Group
