# Supplementary material for: Surgical resection significantly promotes the overall survival of patients with hepatocellular carcinoma: a propensity score matching analysis
Source: BMC Gastroenterol. 2021 May 14;21:220. doi: 10.1186/s12876-021-01807-4 (PMC8120780; doi:10.1186/s12876-021-01807-4)
Supplement: Supplementary file 1 — Additional file 1: Figure S1. Overall survival in patients with BCLC stage 0, A, B, and C disease by Kaplan-Meier analysis. Surgical resection (SR) resulted in significantly higher overall survival than radiofrequency ablation (RFA) and transcatheter arterial chemoembolization (TACE) in BCLC stage 0 (P <0.05) (A). SR resulted in significantly higher overall survival than RFA and TACE in BCLC stage A (P <0.05) (B). SR resulted in significantly higher overall survival than RFA and TACE in BCLC stage A (P <0.05) (B). SR resulted in significantly higher overall survival than RFA, TACE, and other treatment in BCLC stage B (PP <0.05) (C). SR resulted in significantly higher overall survival than RFA, TACE, target therapy, radiotherapy (RTO), hepatic artery infusion therapy (HAIC), and best support care (BSC) in BCLC stage C (P <0.05) (D). [file 12876_2021_1807_MOESM1_ESM.zip › 12876_2021_1807_MOESM1_ESM/Figure S1 20200321R2.pptx]

## Slide 1
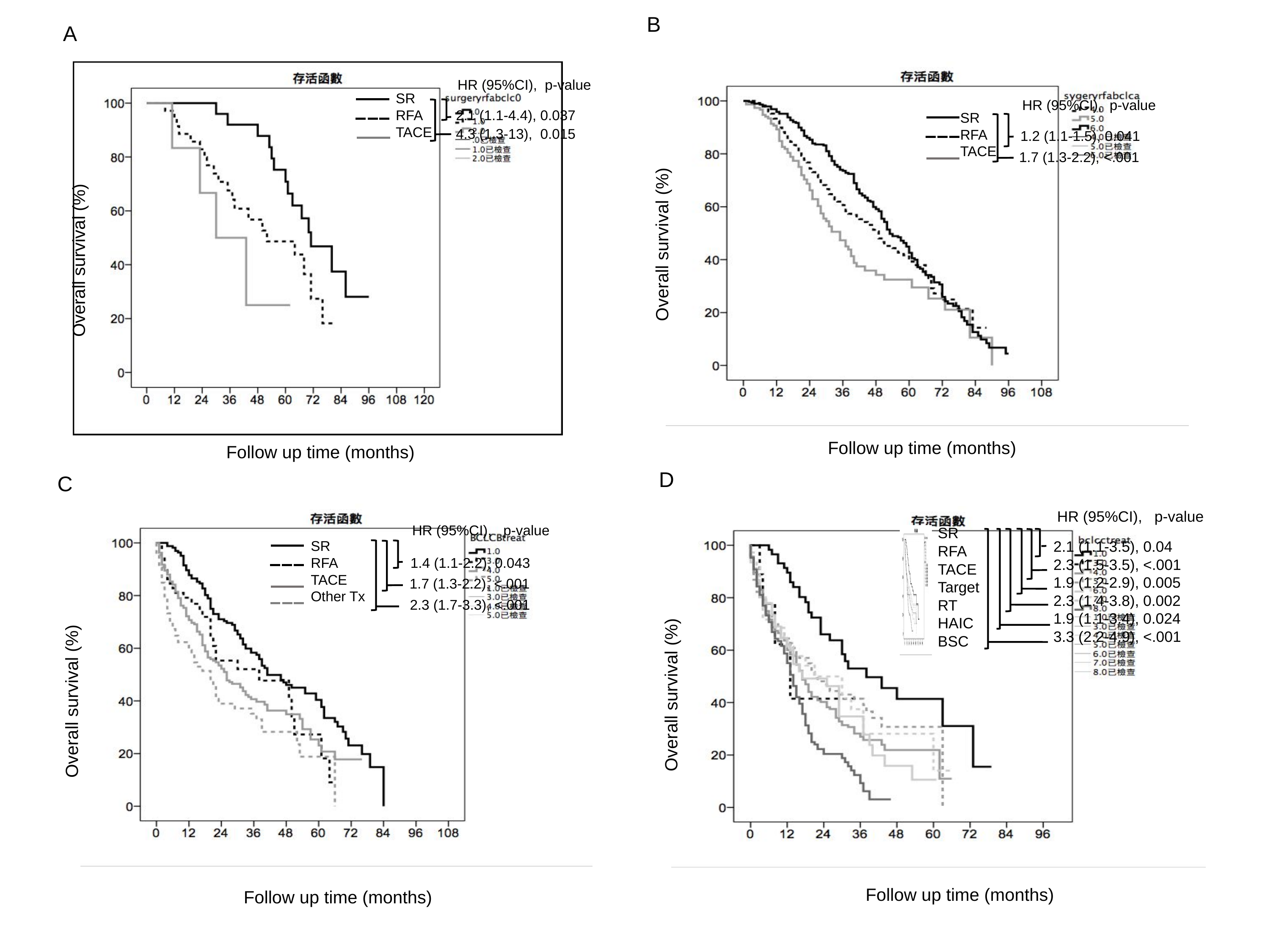

Figure S1
B
Overall survival (%)
Follow up time (months)
 HR (95%CI), p-value
SR
RFA
TACE
1.2 (1.1-1.5), 0.041
1.7 (1.3-2.2), <.001
A
Overall survival (%)
Follow up time (months)
 HR (95%CI), p-value
SR
RFA
TACE
2.1 (1.1-4.4), 0.037
4.3 (1.3-13), 0.015
D
 HR (95%CI), p-value
2.1 (1.1-3.5), 0.04
2.3 (1.5-3.5), <.001
1.9 (1.2-2.9), 0.005
2.3 (1.4-3.8), 0.002
1.9 (1.1-3.4), 0.024
3.3 (2.2-4.9), <.001
SR
RFA
TACE
Target
RT
HAIC
BSC
Overall survival (%)
Follow up time (months)
C
Overall survival (%)
Follow up time (months)
 HR (95%CI), p-value
SR
RFA
TACE
Other Tx
1.4 (1.1-2.2), 0.043
1.7 (1.3-2.2), <.001
2.3 (1.7-3.3), <.001
