# Supplementary material for: Surgical resection significantly promotes the overall survival of patients with hepatocellular carcinoma: a propensity score matching analysis
Source: BMC Gastroenterol. 2021 May 14;21:220. doi: 10.1186/s12876-021-01807-4 (PMC8120780; doi:10.1186/s12876-021-01807-4)
Supplement: Supplementary file 1 — Additional file 1: Figure S1. Overall survival in patients with BCLC stage 0, A, B, and C disease by Kaplan-Meier analysis. Surgical resection (SR) resulted in significantly higher overall survival than radiofrequency ablation (RFA) and transcatheter arterial chemoembolization (TACE) in BCLC stage 0 (P <0.05) (A). SR resulted in significantly higher overall survival than RFA and TACE in BCLC stage A (P <0.05) (B). SR resulted in significantly higher overall survival than RFA and TACE in BCLC stage A (P <0.05) (B). SR resulted in significantly higher overall survival than RFA, TACE, and other treatment in BCLC stage B (PP <0.05) (C). SR resulted in significantly higher overall survival than RFA, TACE, target therapy, radiotherapy (RTO), hepatic artery infusion therapy (HAIC), and best support care (BSC) in BCLC stage C (P <0.05) (D). [file 12876_2021_1807_MOESM1_ESM.zip › 12876_2021_1807_MOESM1_ESM/Supporting figure legend 20210326R2.docx]

**Supporting figure legend**

**Figure S1. Overall survival in patients with BCLC stage 0, A, B, and C disease by Kaplan-Meier analysis.**

Surgical resection (SR) resulted in significantly higher overall survival than radiofrequency ablation (RFA) and transcatheter arterial chemoembolization (TACE) in BCLC stage 0 (*P* <0.05) (A). SR resulted in significantly higher overall survival than RFA and TACE in BCLC stage A (*P* <0.05) (B). SR resulted in significantly higher overall survival than RFA and TACE in BCLC stage A (*P* <0.05) (B). SR resulted in significantly higher overall survival than RFA, TACE, and other treatment in BCLC stage B (*P* <0.05) (C). SR resulted in significantly higher overall survival than RFA, TACE, target therapy, radiotherapy (RTO), hepatic artery infusion therapy (HAIC), and best support care (BSC) in BCLC stage C (*P* <0.05) (D).
